# Exploring Black civil society perspectives of drug decriminalization reforms in the Baltimore context: a participatory action qualitative study

**DOI:** 10.1080/09581596.2025.2532628

**Published:** 2025-07-26

**Authors:** Natalie Flath, Lawrence Grandpre, Corey Shdaimah, Judith Park, Jordan J. White, Brook Kearley

**Affiliations:** a Department of Mental Health, Johns Hopkins Bloomberg School of Public Health, Baltimore, MD, USA; b Leaders of a Beautiful Struggle, Baltimore, MD, USA; c University Maryland School of Social Work, Baltimore, MD, USA; d School of Social Work, Morgan State University, Baltimore, MD, USA

**Keywords:** Decriminalization, structural racism, health equity, antiracist public health practice, participatory action research, drug policy

## Abstract

Drug decriminalization is gaining recognition in academic and policy settings as an intervention to address the increasingly volatile drug crisis and achieve racially equitable drug policy reform in the United States. However, few studies elicit grassroots perspectives of Black communities most affected by legal change. This study explored the perspectives of Black civil society stakeholders on drug decriminalization to inform policy. From 2021 to 2022, racial justice policy advocates and university-based researchers conducted a participatory qualitative study, interviewing twelve key stakeholders a part of culturally responsive, Black-led efforts addressing drug-related harms at the grassroots level in Baltimore City, Maryland. A rapid qualitative analysis revealed that current decriminalization proposals overlook the structural harms of decades of drug criminalization and do not reflect the values that ground communities’ responses to heal and become architects of the solution. Stakeholders recommended the incorporation of financial reparations and investment into a community-led support system that includes Black-owned businesses, harm reduction organizations, and psychospiritual care initiatives. For decriminalization to be effective in urban U.S. contexts, this study emphasizes the need for reforms to be community-led and inclusive of larger U.S. racial justice policy priorities.

## Introduction

Amid overlapping global crisis of mass incarceration, a toxic drug supply, and rising overdose deaths, some public stakeholders propose to counter drug criminalization as a social determinant of health intervention (Park et al., 2020). The U.S. National Institutes of Drug Abuse, the World Health Organization, and the United Nations Office on Drugs and Crime, all call for widespread implementation of decriminalizing drug possession (Csete et al., 2016; Volkow, 2021; Volkow et al., 2017). Although decriminalization is increasingly advocated as a social innovation to redress social and health impacts of incarceration, these policies are rarely conceptualized or led by the communities most impacted (Askew et al., 2022; Greer & Ritter, 2019; Levy, 2018). Most critically, there has been little research on the perspectives of decriminalization policy proposals from Black/African American grassroot communities in urban settings. Little research draws from the economic and cultural systems from which Black communities’ most impacted live, socialize, and work (Hughes et al., 2022). This is a critical gap given US drug criminalization is rooted in a political and social context shaped by systems of racism, which continues to drive criminal legal policies that negatively and disproportionately target Black communities (Alexander, 2020; Black Power Media (Director), 2023; Cooper, 2015; Meeks, 2006). For instance, despite similar rates of drug use across racial and ethnic populations, Black individuals are more likely to be involved in each level of the criminal legal system and more likely to experience adverse drug-related health outcomes (e.g. heightened rates of overdose morbidity and mortality) (ACLU Research Report, 2020; Pew Charitable Trusts, 2022; Rouhani et al., 2024).

Drug policy research often privileges academic and professional stakeholder voices within the purview of major public health organizations such as health departments and universities (Allen, 2004; Hughes et al., 2022). In contrast, Black civil society organizations, which are largely occupied by, serving, and governed by Black people, are missing from drug policy research. Black civil society organizations work to implement local community-driven approaches across multiple sectors outside the government, function within community-based ecosystems of care, and actively work against systemic conditions of racial inequality that shape drug criminalization (Dankwah et al., 2024; McKenzie, 2008; Woods, 1998). This study fills a knowledge gap by eliciting policy perspectives and recommendations on drug decriminalization from Black civil society stakeholders, leaders a part of culturally-responsive Black-led efforts addressing drug-related issues (e.g. substance use disorder, overdose) in Baltimore City, Maryland.

Research is one way to explore knowledge gaps, center-impacted communities, and elevate the themes of social inequality (Hooks, 1996). Research is another way to support the transformative potential of Black-led social movements that bolster Black institutions of communal empowerment and address the community harms of drug criminalization (Varcoe, 2006). Therefore, this study was conducted using a participatory action research approach to 1] open an avenue for impacted Black civil society in Baltimore City to analyze the implications of decriminalization policy initiatives and to 2] inform action. Standard models of participatory research often maintain academic-community partner hierarchies when university researchers control the research theory and design. Our model followed a more robust, co-equal participatory design to enhance the credibility of the findings and align with local racial justice advocacy (Shdaimah & Stahl, 2011; Varcoe, 2006). In partnership with the Director of Research at a grassroots racial justice organization focused on reparations-based drug and criminal legal reforms, the research team collaboratively defined our positionality, theoretical assumptions, research questions, and qualitative methodology. The community-based, non-academic researcher grounded the study in African-centered paradigms, refined the study objectives, led the recruitment of a purposive sample of Black civil society stakeholders, and advised on both the interview guide and interpretation of findings (Grandpre et al., 2023). University-based researchers conducted participant recruitment, interviews, and data analysis.

We used a rapid qualitative analysis (RQA) to inform ongoing drug policy debates, including the Maryland General Assembly’s passage of HB 837, which established a statewide repair and reinvestment fund under cannabis legalization legislation, and the Baltimore City Council’s creation of the Community Reinvestment and Reparations Commission (Bill 23–0353) (Leaders of a Beautiful Struggle, 2023). RQA enabled the timely amplification of community stakeholder perspectives, helping to surface priorities and actionable recommendations to support Baltimore’s grassroots racial justice efforts in criminal legal reform (Hamilton, 2013). By centering voices traditionally excluded from drug policy discourse, this research offers an opportunity to discuss both the limitations and opportunities of current decriminalization proposals and to contribute community-informed policy recommendations.

## Methods

### Context

Various *de jure* decriminalization policies have been experimented with in Portugal (Levy, 2018), the province British Columbia, Canada (Speed et al., 2025) and in Oregon (Good et al., 2025). In the United States, Oregon was the first state to officially trial a *de jure* decriminalization policy in 2021 but subsequently rolled it back (Wire et al., 2025). Around the same time in Baltimore, the State’s Attorney’s Office set national precedence with an unofficial *de facto* decriminalization policy, which paused the prosecution of lower-level drug possession during the COVID-19 pandemic (Rouhani et al., 2023).

This occurred within a racially disparate criminal legal environment that disproportionately and unduly impacts lower-income, Black/African American communities. Although Baltimore City is home to just 9% of Maryland’s population, nearly 40% of the state’s incarcerated individuals are from the city, which is predominantly Black. (Initiative, 2022). A 2016 U.S. Department of Justice investigation found that 89 percent of 100,000 people charged for drug possession were Black, despite declining rates of drug use among them and national trends showing similar rates of illicit drug use across racial groups (Investigation of the Baltimore City Police Department, 2016). They also found that Black Baltimoreans were arrested for drug possession at five times the rate of others, and those arrests rates were 200–500% higher than other similar majority-Black American cities, such as Cleveland, Milwaukee, Detroit and Atlanta. Furthermore, an evaluation of the *de jure* policy found that while drug arrests declined after implementation, racial disparities persisted, with Black Baltimoreans disproportionately affected (Rouhani et al., 2023).

This work comes at a critical time when Baltimore City is facing one of the most severe opioid epidemics in the United States, marked by stark racial disparities (Baltimore City Health Department, 2024). In 2023, the city’s fatal overdose rate was nearly five times the national average. Black male residents aged 60 and older were 4.6 times more likely to die from overdose than their white counterparts.

### Research team and reflexivity

A multidisciplinary research team with diverse professional backgrounds collaboratively conceptualized the study over five months (Grandpre et al., 2023). The team included both university-based (NF, JP, CS, and BK) and a community-based (LG) researcher. Collectively, the team brought expertise in racial justice, social and socio-legal work, criminology, public health, and a range of methodological approaches, including qualitative, quantitative, and African-centered research approaches.

Together we developed a semi-structured interview guide, recruitment criteria, and list of priority stakeholders. LG, a Black man born and raised in Baltimore, serves as Director of Research at *Leaders of a Beautiful Struggle* (LBS), a grassroots think tank that advances the public policy interests of Black people and helps pass numerous racial equity bills, including, but not limited to, police reform and reparations within cannabis legalization. He designed the study’s theoretical framework, developed the research questions, and led the public dissemination of findings to inform Maryland’s cannabis legalization process. LG also facilitated stakeholder recruitment by leveraging LBS’s networks. The university-based team contributed methodological and subject-matter expertise. CS, a white Jewish woman and qualitative methodologist, advised on study design and implementation. BK, a white criminologist and faculty advisor, provided conceptual guidance. NF, a white doctoral trainee in public health, and JP, an Asian woman completing her master’s in social work, conducted the interviews in 2021–2022 and led data analysis. NF also identifies as a member of a community impacted by drug criminalization and disability injustice.

Guided by critical theory and participatory research principles, the team engaged in regular reflexive debriefing sessions throughout all phases of the study to ensure multiple perspectives and critical reflection were integrated into the research process (Birt et al., 2016; Rankl et al., 2021).

### Sampling and recruitment

Stakeholder identification was informed by Black political science scholar Marion Orr’s definition of a Black civil society leader (Orr, 1999). Leaders advance Black interests and values, generate solutions to local challenges, and serve as ‘agents of social reform on issues of economic empowerment, spiritual development, and social and political equality’. We used ‘expert’ purposive and snowball sampling to identify stakeholders who have a leadership history working on Black-led efforts addressing drug-related issues (i.e. substance use disorder, overdose) in culturally responsive ways across Black Civil Society organizations (e.g. faith-based centers, holistic wellness health clinics, drug treatment programs, harm reduction organizations, youth advocacy organizations).

Based on our definition of Black Civil Society, we began by mapping out potential stakeholders involved in predominantly Black-led organizations focused on substance use issues. As a multidisciplinary research team, we collaboratively identified and discussed relevant stakeholders. Our community partner, a prominent organizer affiliated with a racial justice policy think tank, played a key role in identifying stakeholders. Due to his familiarity with community networks often beyond the reach of academic researchers, he identified a wide range of relevant individuals. During the development of our sampling frame and throughout the snowball recruitment process, we applied specific inclusion and exclusion criteria. For instance, we excluded stakeholders working outside of Black Civil Society, such as those employed in government or white-led non-profit institutions. If we received referrals that did not align with these criteria, such as a police officer referral, the individual was deemed ineligible. To ensure diverse perspectives, the research team agreed on a sampling scheme that included both lay and professional experiences (e.g. clinically licensed social worker, grassroots organization). Sample recruitment was facilitated through the credibility of our community research partner. For outreach, LG initiated contact with identified stakeholders, while NF and JP managed follow-up communications, scheduled interviews, and requested additional referrals. Through this process, we identified fifteen potential participants, ultimately interviewing eleven. Although we received multiple snowball referrals (n = 11), only five matched our sampling frame criteria (three community-based drug treatment providers), and just one of these referrals agreed to participate.

The participants were offered a $50 gift card to appreciate their time and effort. Interviews were conducted virtually on the secure online videoconferencing Zoom platform, lasted approximately an hour and a half, and were audio-recorded and auto-transcribed on the Zoom platform. NF and JP then cleaned the transcripts to ensure they reflected interviews verbatim.

As shown in Table 1, we interviewed twelve Baltimore-based Black civil society stakeholders who served roles as faith-based leaders, community-based holistic wellness and drug treatment professionals, scholars specializing in African-Centered approaches to substance use from Historically Black College and Universities, youth advocacy program organizers who work with youth involved in the drug trade, community-based youth violence and drug prevention elders working with families with substance use issues (e.g. Rites of Passage), a Black harm reduction community organizer, and community-based clinical social work therapists serving people and families with substance use disorder. Stakeholders acknowledged they were informed by African-Centered, Black radical, and/or indigenous healing traditions. Therapists integrated spiritual traditions (e.g. plant medicines, acupuncture), worked independently or with Black-led community-based organizations, and had experience working in both community, healthcare, and criminal justice settings. For example, one of our stakeholders is a founder of Baltimore’s first community-based harm reduction and drug treatment clinic with a focus on cultural modals of holistic healing (e.g. acupuncture, group therapy), which still exists today in a predominantly Black low-income neighborhood.

### Ethical considerations

Verbal informed consent outlining study procedures and voluntary participation was obtained and recorded at the start of the interview. The nature of the stakeholder study allowed for an oral consenting process. Ethics approval was obtained by University of Maryland Baltimore’s Institutional Review Board (HP-00098974) and adheres to the Declaration of Helsinki. In this study, the participants are identified using pseudonyms to protect confidentiality.

### Data collection and analysis

The interview guide consisted of open-ended questions and probes for clarification. The questions focused on participants’ perspectives on the causes of substance misuse and overdose, relationships between community and non-community-based drug prevention and treatment organizations, drug decriminalization, and policy and practice recommendations for drug policy advocacy, which the latter is the focus of this study (see supplementary material for the interview guide).

We conducted a qualitative rapid analysis based on Hamilton and colleagues’ guidelines (Hamilton, 2013). Rapid analysis is a deductive, in-depth qualitative methodology that produces rapid, relevant, and responsive qualitative findings for applied use (Taylor et al., 2018). Rapid analysis was performed simultaneously with data collection, allowing for systematic and expedient interpretation of relevant themes to inform ongoing state-level drug policy efforts (St. George et al., 2023). To enhance study rigor, the research team applied a collaborative approach for rapid analysis (Richards & Hemphill, 2018). During data collection, the interviewers (NF and JP) met after each interview, and the working group met after two interviews, maintaining an organized audit trail of memos and meeting notes. We documented transparent decisions regarding interview-guide modifications, sampling decisions for subsequent interviews, and sufficiency monitoring for understanding the depth and completion of our sampling.

Given the time-sensitive nature of this action research, designed to inform an upcoming legislative session, and our focus on a knowledge base not traditionally tapped by academic researchers, we aimed to conclude data collection once we illuminated a robust and diverse picture of perspectives from Black Civil Society stakeholders working on substance use issues with culturally grounded approaches. We concluded data collection once recurring themes emerged across a diverse participant sample, including generational variation, indicating our data reached sufficiency (Staller, 2021). Sufficiency was also determined in two other ways. First, each interview offered rich, in-depth reflections that provided sufficient data to illuminate themes and answer our questions. Second, there was notable variation in how stakeholders interpreted the impacts of and solutions to drug criminalization. While most rejected criminalization, two stakeholders offered contrasting views, seeing drug courts as a potential pathway to treatment. These negative cases demonstrated the diversity of perspectives captured within the sample. The depth of the interviews also contextualized these divergent views, linking them to broader cultural values that emphasized care and inclusion for people with substance use disorders as valuable community members. Together, the richness and variation across stakeholder experiences provided a strong rationale for concluding data collection.

At the beginning of the interview process, the lead author (NF) created an ‘interview summary’ template in Microsoft Word to compile non-interpretative summary points for the two key questions of interest: 1) perspectives of decriminalization and 2) policy recommendations. After each interview meeting, NF read through each transcribed interview and meeting note to organize key quotes and salient themes separated by the two questions in the matrix summary. After data collection was complete, the full research team reviewed the matrix summary, established a consensus on themes, and selected final illustrative quotes.

## Results

Black civil society in Baltimore City, who are a part of culturally grounded, community-rooted efforts addressing drug-related harms (e.g. substance misuse, overdose) raised two key perspectives on drug decriminalization and offered two recommendations for future policymaking. Stakeholders shared that 1) decriminalization will not change the larger structural conditions faced by communities targeted by the War on Drugs; and 2) current decriminalization efforts do not reflect the values that ground communities’ responses to heal and become architects of their own solutions. They recommended to 1) require community investments, specifically racial reparations, to amend the larger structural harms experienced by communities targeted by the War on Drugs and 2) to ensure decriminalization reforms are community-led.

### Drug decriminalization will not change the larger structural conditions faced by communities targeted by the war on drugs

Stakeholders reflected on both the promise and limitations of decriminalization. They noted its potential to reduce the disenfranchising effects of racialized drug policing and support the return home of incarcerated community members. They also viewed decriminalization as a potential catalyst for healing through the reunification of individuals and family members after decades of disruption from the ‘war on drugs.’

However, stakeholders expressed concern that removing arrests for low-level drug possession alone does not address the broader structural conditions that drive racialized drug criminalization experienced by Black communities in Baltimore and worried about the limits of the policy’s intended impacts on racial equity. Stakeholders identified the core policy issues as rooted in the structural conditions of poverty and over-policing in predominantly Black neighborhoods, all of which exacerbate and reinforce drug criminalization and a reliance on the drug trade to meet basic financial needs. Stakeholders agreed that current decriminalization reforms do not address the social and economic consequences of longstanding drug criminalization, which decimates community structures and leaves communities with limited options for legal employment, increases engagement in the illicit street economy, and heightens the risk for other drug-related harms (e.g. overdose). One community-based mental health provider recalled his own experience and summarized this:

It’s tough for me because I live in a community, and I have a child, and I don’t think that everyone should just be out here, selling drugs. I’m not anti-criminalization of substances, but we can’t criminalize and not improve the conditions that facilitate criminality at the same time. It places the blame on people, usually people of color, people who are poor, people who have less education. It doesn’t fall on the powers that be and the people with money. (Calvin)

Stakeholders pointed out that minor amendments proposed by decriminalization, such as reconceptualizing levels of thresholds of drug possession, ignore the structural effects of decades of racialized drug criminalization. They were concerned that if these cyclical structural conditions were not recognized and addressed, they would worsen in post-decriminalized Baltimore. For example, a African-Centered scholar and Baltimore elder shared an example of how mainstream decriminalization may contribute to street-based violence if the precarious circumstances that place people in positions to engage in the drug trade for economic survival are not central to policymaking:

[T]he big business of the drug trade provides opportunities for primarily young Black men who are not gonna be a part of the so-called primary labor market... to make money, but it also obviously places them at risk to be incarcerated and murdered... The opportunities that vulnerable people have in the streets to make money is a way to also keep them cool and calm... if you don’t have the services and the opportunities there, to rebuild and help the people, they will become more desperate, and they are not going to suffer peacefully. They’re going to turn that anger inward and outward, and I would say there would be more crime on the street. (Jay)

Similarly, one youth advocate expressed concern that decriminalization is not applicable to minors, opening a loophole that may worsen the structural conditions of Black youth and their families. He discussed how decriminalization within other liberal drug policy reforms, such as cannabis legalization, risks the continued criminal surveillance and racialized social control of Black youth under 18 who engage in drug trade to meet financial household needs.

The problem is in poor communities, like Black people’s communities traditionally, there’s a huge and urgent need for additional streams of income. And there’s a pretty well-documented history of kids taking up that type of ownership over contributions to the household space. If you are a kid with no formal training, no legitimate connections to larger money or politics or anything else, you have what’s around you so, traditionally, selling a little bit of weed is most people’s introduction into being able to make money… there could be potentially a bunch of kids, who while this product is legal both medically and recreationally, because they are trying to provide for their families in the same way that these White cannabis companies are, will continue to be arrested, and continue to be engaged in the juvenile justice system. (Milton)

In the absence of politically tangible solutions to address structural issues rooted in anti-Black structural racism, stakeholders were concerned that decriminalization reforms could be packaged into other forms of criminal legal surveillance. Given the context of the hyper-criminalization of Black Baltimore residents and where drug possession and distribution are still clandestine, we heard concern that even with decriminalization of small drug quantities, police would still retain broad discretion to charge individuals with intent to distribute. This, they warned, would continue the systematic surveillance and incarceration of predominantly Black neighborhoods and undermine the stated goal of advancing racial equity.

### Decriminalization does not recognize the values that ground communities’ responses to heal and be architects of their own solutions

Another key concern among Black civil society stakeholders was the lack of meaningful community participation in shaping decriminalization policies. They criticized how professionalized public health leaders within established ‘anchor institutions’ often hold disproportionate power to define the terms of intervention, speak on behalf of Black civil society, and influence political, economic, and real estate decisions. As a result, trusted community leaders, such as youth mentors, harm reduction workers, and faith-based leaders, are excluded from policymaking, despite their frontline roles in addressing the intergenerational harms of substance use and criminalization.

This was recognized as part of a broader pattern in which drug policy efforts have excluded impacted communities from developing their own responses to drug use and criminalization. Not only was this exclusion viewed as a major barrier to accepting the policy, but it also raised several concerns. Diversion programs often refer individuals to services outside of community-led organizations, which may conflict with culturally grounded values of healing and recovery. For example, mainstream programs like 12-step models were seen as isolating and misaligned with principles of community integrity and mutual support. Stakeholders also contributed the policymaking exclusion to the absence of Black-led institutions within the harm reduction and drug treatment ecosystem. As a result of this, they noted that the concentration of services outside of Black community governance reinforces racialized economic disparities by limiting opportunities for Black civil society and perpetuating power imbalances and cycles of poverty. Milton provides the following example:

Let’s say my uncle gets locked up for possession because he had some dope on him. Let’s say he did get a court date…and the judge said, ‘Alright, you either got to do 90 days or go to a drug treatment program.’ It’s going to be this list of pre-approved vendors that you got to go through…you don’t know who works for who and what’s going to work. Even though everybody’s auntie and grandmother can cook, nobody’s ever afforded the ability to go cook in the school kitchen because there’s already a set of providers [outside of the community]. **Because usually again, systems of White supremacy and anti-Blackness don’t allow the thought that Black people can be the architects of their own solutions.** [emphasis added] (Milton)

Drawing on their experiences within the mainstream drug treatment and harm reduction landscape, they also noted that investments in Black communities are often temporary, and this fails to address, if not exacerbates, underlying structural conditions. Another community-based mental health provider and former administrator in the drug treatment system described how financial incentives in the system not only also reinforce racialized economic disparities by anchoring service control in the criminal legal system, but discourages healing and recovery among people who use drugs:

[In] drug court programs... money is driven by grants. Their incentive again is money, which means they need a constant influx of people coming into drug court. [People] have to graduate from drug court, which means you have to graduate from the program, but primarily the incentive is not the person’s improvement, it’s how do we keep our jobs at the court, the prosecutor, the judge. I’ve seen it first-hand, internally the criminality of the system itself. People are buying and selling clients to each other, literally seen trading clients among court officials working together to make sure that only certain programs receive clients because they are affiliated with one program and then funnel clients from that program to get clients only from them. (Calvin)

### Require reparations to amend the larger structural harms experienced by communities targeted by the war on drugs

The stakeholders’ most consistent recommendation was to invest in the social conditions of Black communities most targeted by the War on Drugs. To do this, they emphasized supporting culturally grounded systems of care and economic development, such as Black-owned businesses, harm reduction organizations, and psychospiritual care healing spaces, and investing in a broader community ecosystem centered in cultural values, local talents, and community institutions.

The programs of the future will be funded better, but also will take that funding and begin to see the importance of an integral healing where we touch in on all the available areas of a person’s development, which is inside us, as individuals, our psychospiritual selves, inside of us as groups, our culture, the outside of us as individuals, which is our bodies, and the outside of all of us, which is our society and our social systems. (Calvin)

Investments in collective care systems consistently pointed to Black business and economic development that serve to meet social needs, such as housing, health and nutrition. A holistic drug treatment consultant pointed out the following.

Reallocate money. Put behind local businesses, especially local businesses… that have a holistic kind of a bend. How does the money stay in the community? Local businesses that create ecosystems, right? So that’s food, and housing. (Amy)

Similarly, a Black transgender women and harm reduction grassroots organizer reiterated and called for dedicated funding to strengthen community-based infrastructure outside the traditional public health system:

Reparations [should be] going directly to Black and brown people and not into big organizations [outside of Black civil society] … The funds get lost after they funnel into the community [through outside organizations]. I want us to invest in Black and Brown businesses and harm reduction organizations and beef up our communities with things for people to do other than do drugs. Like neighborhoods to be well-rounded, with parks and no food deserts. (Monica)

Reinvesting in communal ecosystems of care was also seen as a vital decriminalization strategy to heal the psychospiritual harms shaped by racialized drug policing and incarceration, affecting both people who use drugs and their broader communities. Stakeholders described how drug criminalization deepens the loss of culture and connection in Black communities, fueling psychospiritual pain and drug-related harms (e.g. substance use disorder). This pain stems from the traumatic experiences of losing loved ones to the criminality tied to drug use, overdose death, and engagement in the illicit drug economy. One community-based mental health provider who also specializes in therapy for grassroots racial justice organizers stressed the importance of addressing this pain through psychospiritual approaches grounded in political awareness of anti-Black oppression.

Decriminalization would be easier if we had more voices of folks that had some critical consciousness experience where they had the ability to see themselves in their own life, see it, alongside what is happening politically, and then begin to advocate and be supported by community, so that they could be in the face of, and be supported collectively, and transforming everything that we think about when we think about why people use drugs. They use drugs because they are trying to connect to spirit or are in deep, deep, pain and trying to numb that pain. How can we create a space where everyone feels like they don’t have to escape through a substance to heal their pain? (Donna)

Along with other stakeholders, Donna emphasized the need for decriminalization proposals that are accompanied by investments in systems that build supportive community environments, rather than advocating solely for incremental criminal legal reforms. She reiterated that simply decriminalizing personal drug possession does not address the psychospiritual harms experienced by people who use drugs, their families, and communities, and echoed other stakeholder recommendations by highlighting examples of collective care aimed at restoring cultural connection in Black communities in Baltimore. These included, Black-led community mental health services grounded in African spiritual traditions, such as dance, gardening, connection to nature, and guided meditations, as well as other Indigenous practices outside of Eurocentric medical models, like storytelling with medicine wheels and detoxifying acupuncture. Faith-based centers, particularly Black churches, were also identified as a central sites of psychospiritual healing, offering culturally affirming care, and spiritual support:

Another indigenous way, and we need to use more of this, a major institution in the Black community, is the Black church.... Positive transformation in the Black community has been through the church, so you’re not using everything in your arsenal if you’re not using the church. Churches across the nation have mental health and substance use services. These services integrate Eurocentric and African-centered flavors around spirituality and collective responsibility. A component of the treatment model is demonstrating how an individual African American particular life can be used, not just to advance oneself, but to help advance the broader collective African American community to advance the race. (Jay)

#### Ensure decriminalization reforms are community-led

Stakeholders underscored the need for more grassroots expertise in the design and implementation of decriminalization proposals and offered a range of recommendations to ensure reforms are developed and implemented by Black civil society. Some advocated for meaningful community participation in policy workgroups, while others called for shifting policy development and harm reduction efforts completely toward grassroots Black institutions, explicitly excluding predominantly white-led private ones. They emphasized this as a necessary component for preventing decriminalization policies from expanding the reach of police and intensifying the systematic surveillance of people who use drugs in Black neighborhoods. State and private agencies often collaborate with law enforcement to design drug-related interventions, raising concerns that policing under decriminalization would shift from arrests to state-managed treatment surveillance. A grassroots harm reduction organizer encapsulated these concerns by offering concrete recommendations to ensure accountable, community-led implementation of decriminalization efforts:

I would love for participants to own and run them [drug treatment and harm reduction services], but I feel like that’s never going to happen. White people from Johns Hopkins University are going to own and run them. But my hope is that we start mentoring people within the community to take over these executive director and community organizing positions. (Monica)

## Discussion

This study extends the current decriminalization debate by eliciting the participatory knowledge of Black civil society stakeholders in Baltimore City, a city with a deeply entrenched legacy of racially discriminatory drug enforcement. Similar to other research eliciting the perspectives of people who use drugs globally, stakeholders highlighted the void of communal values in decriminalization discourse as both a consequence and cause of the exclusion of impacted community voices in policymaking (International Network of People who use Drugs, 2021). Stakeholders reflected on the opportunities and limitations of decriminalization, echoing findings from emerging studies that center impacted community perspectives, such as advocating for policies that better bolster investments in community-based, resilient systems of care. Building on this literature, stakeholders stressed the need for a paradigm shift that addresses the sociocultural and structural consequences of decades-long drug criminalization experienced by Black communities in Baltimore and envisions decriminalization as part of a broader racial justice agenda, inclusive of police accountability and reparations for the War on Drugs (Flanigan & Freiman, 2023; King & Page, 2018).

These findings contribute to a growing body of literature documenting community-based perspectives on the limitations of decriminalization when implemented without structural reforms (Levy, 2018; Speed et al., 2025; Ziv et al., 2023). Specifically, the concern that decriminalization alone does not address the structural conditions faced by communities targeted by drug criminalization and may therefore fall short of its intended public health goals, echoes research rooted in Black community perspectives on how to address the racialized overdose crisis (Banks et al., 2023; Dankwah et al., 2024; Lopez et al., 2022; Seo et al., 2023). Prior studies with Black church leaders and grassroots health workers describe overdose as a symptom of entrenched social and structural inequities, including the lack of intra-community financial resources, and disproportionate exposure to racialized policing. Consistent with our findings, these studies recommend investing in Black communities, strengthening networks of care, and embedding mutual aid into overdose prevention strategies to address the aforementioned structural barriers.

Similar concerns about decriminalization have emerged in studies with people who use drugs in British Columbia, Sydney (Australia), and Oregon, where many argue that under continued systems of criminalization, incarceration, and social exclusion, decriminalization remains limited in impact, particularly among people who use drugs with intersecting marginalized identities (Dertadian & Sentas, 2025; Good et al., 2025; Pamplin et al., 2023; Xavier et al., 2022). A study in Oregon found that despite the enactment of decriminalization, PWUD continued to face unpredictable and, at times, retaliatory policing. (Good et al., 2025) Unhoused individuals remained especially vulnerable, and widespread mistrust of law enforcement continued to be barriers to accessing police-mediated services.

Contemporary decriminalization is often framed as a singular overdose prevention strategy that will advance health equity through a social determinants of health lens (Dasgupta et al., 2018; Park et al., 2020). However, both our stakeholders and a review by the International Network of People Who Use Drugs have argued that the standard model falls short (Levy, 2018). It often overlooks how public health systems remain entangled with carceral institutions, allowing the criminalization of drug use to persist in new forms. Stakeholders emphasized that structural drivers, such as the role of interlocking public institutions (e.g. public health and legal systems) in sustaining racialized hyper-criminalization, are rarely addressed in either the conception of the problem or the development of solutions.

Prior studies echo these critiques. Research in Black communities has shown that harm reduction efforts embedded within public health-police partnerships reinforce racial hierarchies, exclude Black cultural frameworks, and perpetuate drug-related harm (Lopez et al., 2022; Woods, 1998). Similarly, qualitative research with people who use drugs engaged in social services reported widespread skepticism about the impact of current drug reforms amid systems with entrenched social stigmas (Askew et al., 2022; Greer & Ritter, 2019; Levy, 2018). Michaud et al. (2024) describe this phenomenon as ‘adaptive criminalization,’ noting how Vancouver, Canada’s 2023 decriminalization rollout extended legal surveillance into health and social services, subjecting marginalized communities to increased criminal legal surveillance when accessing care (Michaud et al., 2024).

Echoing global concerns from people who use drugs, stakeholders in this study emphasized the limitations of decriminalizing small quantities of drug possession (International Network of People who use Drugs, 2021; Xavier et al., 2022). While this existing literature notes that thresholds are problematic due to the blurred line between use and distribution, participants offered a novel, deeper critique. They argued that standard decriminalization models fail to account for the economic realities of individuals who use and sell drugs for survival amid racialized economic inequality. In cities like Baltimore, structural forces such as deindustrialization, gentrification, and mass incarceration have reduced employment opportunities and driven the expansion of informal economies (Agar & Schacht Reisinger, 2002; Schiele, 2013). A mixed-methods study with West Side Chicago residents similarly identified the illicit drug economy as a critical means of meeting basic needs (Theodore, 2021). Therefore, stakeholders expressed concern that, standard approaches overlook the needs of those involved in informal street economies, limiting the potential of decriminalization to advance racial equity. They posited that in the absence of broader economic reform in Black neighborhoods that are disproportionately targeted by drug policing and incarceration, decriminalization may lead to increased policing of poverty-based offenses, such as loitering and unlicensed street vending, and further criminalize Black individuals working in the illegal drug market for financial survival. This is a phenomenon known as net-widening (Michaud et al., 2024). Research shows that post-decriminalization, policing often shifts from drug possession to other low-level offenses (Kozlowski et al., 2019; Michaud et al., 2023). For example, in one Maryland county, marijuana decriminalization in 2014 was followed by a rise in misdemeanor traffic charges that disproportionately affected Black residents (Kozlowski et al., 2019).

To proactively address these concerns in future policymaking, stakeholders in our study called for decriminalization models that foreground racial justice, invest in community-led solutions, and fully disentangle care from law enforcement. One example is Newark’s Community Street Team (NCST), a Black-led, evidence-based, community-grounded model that integrates harm reduction and anti-violence services while explicitly removing police from public health responses (Leap et al., 2020). Additionally, this collection of interviews provide us with tangible suggestions for coupling decriminalization efforts with policies that 1) invest in economic equity for Black workers engaged in an informal economies and 2) challenge racialized systems that perpetrate social vulnerability and police targeting (Bass, 2001). In Baltimore, one promising step in this direction is the ongoing strengthening of civilian oversight to improve police accountability and prevent over-reach (Costello, 2024).

This research is timely, as decriminalization proposals are facing political pushback across the United States (Faiola & Martins, 2023). A central critique is the lack of investment in health and social services to replace arrest and incarceration (Baker, 2024; Laqueur, 2015; Ziv et al., 2023). This gap has been cited as a key barrier to the success of Portugal’s model, the first country to implement decriminalization (Laqueur, 2015). In Portland, Oregon, the mayor acknowledged the absence of a behavioral health infrastructure as a major factor in the 2024 rollback of the pilot, which initially lacked funding provisions for treatment in the original bill (Knopf, 2020). The public also blamed the policy for increases in homelessness and overdose, prompting a return to punitive drug measures. Similar concerns were raised by stakeholders in this study. They reiterated that without meaningful investment in community-led mental health and drug treatment services, decriminalization alone will remain insufficient, and potentially unacceptable, for Black communities. Moreover, this study comes at an opportune time to inform real-time funding streams, such as the Community Reinvestment and Reparations Commission and the Opioid Restitution Funds advisory board, in support of community-driven initiatives to prevent overdose (Leaders of a Beautiful Struggle, 2023; Opioid Restitution Fund, 2024).

### Limitations

This research initiated a repository of grassroots perspectives on local decriminalization efforts in Baltimore. We used a participatory methodology to contextually ground a communal understanding of drug policy reform and inform current legislative efforts. However, this study has limitations. First, Black civil society is diverse, and we may not have captured all perspectives. Not only does power asymmetry manifest in the context of an academic and non-academic community-based research partnership, it may also act as a barrier to the inclusion of participants outside our connections. Connecting personal stories to larger narratives of liberation and structural violence can be challenging, as those seen as credible messengers may favor those with educational and professional privilege or those with clear and consolidated influence. While our sample includes locals engaged in African-centered healing and members of the Nation of Islam, voices from diversified faith communities, such as Muslim Imams, may offer unique insights that we did not capture. Given the exploratory nature of rapid analysis for dissemination, future research warrants further exploration of perspectives to generate a more robust theoretical understanding of decriminalization using grounded theory methods. Also, more research that devolves power dynamics is needed for envisioning and developing emancipatory drug policy making. Second, our research is context-specific and cannot be fully transfered to other places, which warrants continued research among grassroots leaders across similar urban settings. Despite these limitations, the participatory study enhances the credibility of the findings by positioning theoretical assumptions in cultural research paradigms and tackling social desirability bias inherent in the standard model of qualitative academic research.

## Conclusion

This study underscores the importance of grounding decriminalization efforts in liberatory, cultural frameworks and offers valuable lessons for public health. By centering traditionally marginalized perspectives through a participatory research process, we shifted the locus of knowledge production from academic to grassroots leaders who actively analyze and push back against systemic racism in their everyday work. In doing this, we learned new lessons about how to advance racial equity. We applied an approach that serves as a future model for more equitable drug policy development, and we learned new ways to support ongoing local efforts working to channel investments in Black communities most affected by the drug war.

## Supplementary Material

Supplementary Material

Supplemental data for this article can be accessed online at https://doi.org/10.1080/09581596.2025.2532628.

## Figures and Tables

**Table 1.** Participant pseudonyms and descriptions of Black civil society stakeholders, Baltimore City, Maryland (n = 12).

Stakeholder pseudonym	Community identified role	Age group*	Racial identity	Gender identity

Milton	**Youth advocate and mentor,** afterschool CBO^^^ program director serving youth and families with involvement in the street economy, informed by the Black liberation movement.	Young Adult	Black	Man
Jackson	**Holistic Community-Based Therapist & Healer,** clinical social worker who runs an independent Black-owned community clinic incorporating integrative holistic healing (Reiki, yoga) and hosting African-Centered and Indigenous healing workshops.	Adult	Black	Man
Jay	**Scholar,** on African Centered approaches to substance misuse at Historically Black College and University.	Elder	Black	Man
Olusola	**Community-based organizer and African-Centered cultural worker,** supporting Rites of Passage programs for youth and families affected by drug issues for 40 years.	Elder	Black	Man
Amy	**Holistic addictions consultant and acupuncturist,** for community-based addiction recovery models, emphasizing community repair and healing, and was an early member of one of the first Black-led, grassroots drug treatment organizations based in a majority-Black neighborhood.	Adult	White	Woman
Agnes	**Faith-based church leader,** (pastor) serving neighborhood members and families experiencing a substance use disorder.	Elder	Black	Woman
Rashard	**Youth advocate and mentor,** founder of youth-run student CBO focused on racial justice organizing to reform education systems and build Black-led organizations, informed by an African sovereigntist worldview. Community organizer and church leader.	Young Adult	Black	Man
Charles	**Holistic Community-Based Therapist & Healer,** working to oversee and administer substance use care since the 1970s in leadership roles in both community-based and medical settings. Now is a nursing professor specializing in African-Centered substance use research and practice.	Elder	Black	Man
Donna	**Holistic Community-Based Therapist & Healer,** clinical social worker/therapist serving Black women and activists, integrating African-Centered healing traditions. Social work doctorate and grassroots activist.	Adult	Black	Woman
Monica	**Grassroots harm reduction organizer**, working in a community-based harm reduction organization.	Adult	Black	Transgender Woman
Tabatha	**Holistic Community-Based Therapist & Healer,** clinical social worker and integrative healer specializing in spiritual traditions (e.g. plant medicine, meditative practices), experienced in community-based and criminal justice settings.	Elder	Black	Woman
Calvin	**Holistic Community-Based Therapist & Healer,** clinical social worker operating an independent Black-owned mental health clinic, specializing in substance use disorder and psychedelic integration therapy, meditation, and nature-based healing, informed by an African-Centered worldview and Buddhist practice.	Adult	Black	Man

Notes.

* Young Adult: ~20–30, Adult: ~30–55, Elder: ~55+.

^ CBO community-based organization.

Note: demographic information was assigned to the best of the knowledge of the research team.

## Data Availability

The participants of this study did not give consent for their data to be shared publicly. Given ethical considerations of the sensitive nature of the research and identifiability of the sample, supporting data is not available.
