# Supplementary material for: Exploring Black civil society perspectives of drug decriminalization reforms in the Baltimore context: a participatory action qualitative study
Source: Crit Public Health. Author manuscript; Available in PMC 2026 Jun 16. (PMC13268585; doi:10.1080/09581596.2025.2532628)
Supplement: Supplementary Material [file NIHMS2166335-supplement-Supplementary_Material.docx]

Supplementary Material

| Supplemental Table 1. Interview Guide Domains and Example Questions | |
| --- | --- |
| Semi-Structure Interview Guide Domains | Questions |
| Community-based role | Can you tell me about the work that you do in your community?  What values inform your work?  Probe: Religious, spiritual, cultural, historical  Probe: Have they changed overtime |
| The root causes of addiction and adverse drug outcomes | Can you tell me about how drug use impacts your community? Probe: For people using drugs, for families, for the neighborhood more generally, for business owners?  What do you think are some “root causes” of drug addiction? Probe: How does the larger environment of history and culture impact drug addiction and overdose? |
| Perspectives on community-based and state-sponsored public health and treatment programs | Please tell us about what resources and organizations exist within your community to address the impacts of drug use and drug addiction and how they work.  Probe: Family, friends, businesses, churches, etc. (who runs them, who funds them, how do people access them, what services do they provide-financial, housing, addiction care, emotional)  Probe: How have you seen these change over time? Legislative, social, cultural, political factors  Probe: In what ways do you see these resources/organizations fostering or coming at odds with the community values you mentioned earlier?  What about organizations and resources that provide supports but are from outside the community?  Probe: Government programs, universities, non-profits (who runs them, who funds them, how do people access them, what services do they provide)  Probe: How have you seen these change overtime?  Probe: In what ways do you see these resources/organizations fostering or coming at odds with community values you mentioned earlier?  How do the resources/organizations outside the community compare with those within?  Probe: How do they interact? Work together? How are they at odds? |
| Policy and practice recommendations | Can you give me some examples of what society would look like if the “root causes” of drug addiction you mentioned previously were addressed?  Probe: Given these concrete examples, what would the role of organizations/resources within and outside your community look like in society? Mission, purpose, origin, philosophy, structural relationships of organizations, leadership |
